# Lived Experience in New Models of Care for Substance Use Disorder: A Systematic Review of Peer Recovery Support Services and Recovery Coaching

**DOI:** 10.3389/fpsyg.2019.01052

**Published:** 2019-06-13

**Authors:** David Eddie, Lauren Hoffman, Corrie Vilsaint, Alexandra Abry, Brandon Bergman, Bettina Hoeppner, Charles Weinstein, John F. Kelly

**Affiliations:** ^1^Recovery Research Institute, Center for Addiction Medicine, Massachusetts General Hospital, Harvard Medical School, Boston, MA, United States; ^2^Department of Psychiatry, Massachusetts General Hospital, Harvard Medical School, Boston, MA, United States

**Keywords:** peer recovery support services, recovery coaching, peers, substance use disorder, addiction

## Abstract

Peer recovery support services (PRSS) are increasingly being employed in a range of clinical settings to assist individuals with substance use disorder (SUD) and co-occurring psychological disorders. PRSS are peer-driven mentoring, education, and support ministrations delivered by individuals who, because of their own experience with SUD and SUD recovery, are experientially qualified to support peers currently experiencing SUD and associated problems. This systematic review characterizes the existing experimental, quasi-experimental, single- and multi-group prospective and retrospective, and cross-sectional research on PRSS. Findings to date tentatively speak to the potential of peer supports across a number of SUD treatment settings, as evidenced by positive findings on measures including reduced substance use and SUD relapse rates, improved relationships with treatment providers and social supports, increased treatment retention, and greater treatment satisfaction. These findings, however, should be viewed in light of many null findings to date, as well as significant methodological limitations of the existing literature, including inability to distinguish the effects of peer recovery support from other recovery support activities, heterogeneous populations, inconsistency in the definitions of peer workers and recovery coaches, and lack of any, or appropriate comparison groups. Further, role definitions for PRSS and the complexity of clinical boundaries for peers working in the field represent important implementation challenges presented by this novel class of approaches for SUD management. There remains a need for further rigorous investigation to establish the efficacy, effectiveness, and cost-benefits of PRSS. Ultimately, such research may also help solidify PRSS role definitions, identify optimal training guidelines for peers, and establish for whom and under what conditions PRSS are most effective.

## Introduction

Substance use disorder (SUD) is one of the most pervasive and intransigent clinical and public health challenges facing the United States (Office of the Surgeon General, 2016). While many who meet criteria for SUD are able to achieve remission without formal treatment (Cunningham and McCambridge, 2012; Kelly et al., 2017), many millions of affected individuals require some combination of acute care, medical stabilization, long-term recovery management, and recovery support services to sustain remission, akin to the care of other chronic health conditions such as diabetes and hypertension (McLellan et al., 2000). There is evidence that such multifaceted, long-term care models for SUD are helpful (Dennis et al., 2003; Scott and Dennis, 2009).

Existing health-care and treatment models, however, are often not structured in ways that facilitate treatment engagement, and linkages to services that can support long-term remission of SUD (McLellan et al., 2000; White and Kelly, 2011). To begin to address this care gap, many healthcare institutions have begun to implement peer recovery support services (PRSS) to help initiate and maintain patients' engagement with SUD treatment and other recovery support services, and mitigate relapse risk.

First arising in the 1990s, PRSS for individuals with SUD emerged from a variety of predecessors inside and outside of the addiction field. “Patient navigator” models have played important roles for several decades in the professional coordination of care for chronic medical conditions such as cancer (e.g., Robinson-White et al., 2010; Freeman, 2012), and later included peers with lived experience to aid engagement (e.g., Giese-Davis et al., 2006). Such navigator models have also been developed in the care of individuals with severe mental health conditions (e.g., Corrigan et al., 2017). There is also a long tradition of community-based 12-Step mutual-support (e.g., “sponsors”), that can provide free ongoing recovery monitoring and management using peers with lived experience, though this class of peer support should not be conflated with more structured PRSS that are increasingly being incorporated into clinical settings and can support multiple pathways to recovery.

In the SUD field, PRSS are most often peer-driven mentoring, education, and support ministrations delivered by individuals who, as a result of their own experience with SUD and SUD recovery, are experientially qualified to support peers with SUD and commonly co-occurring mental disorders. These services represent a new category of specialized resources that are not formal treatment and not mutual-help, which offer support as well as linkage to traditional addiction treatment and mutual-help recovery programs (White and Evans, 2014). These PRSS roles emphasize respect for the diverse pathways and styles of recovery, and stress the need for long-term continuity of recovery support through mobilization of personal, familial, and community help (Valentine, 2010; White, 2010). They can be delivered through a variety of organizational venues and a variety of service roles including paid and volunteer recovery support specialists.

SAMHSA has previously defined PRSS as a peer-helping-peer service alliance in which a peer leader in stable recovery provides social support services to a peer who is seeking help in establishing or maintaining their recovery (SAMHSA, 2009). This broad definition provides a useful starting point that may help guide PRSS practice and research, however, it doesn't describe the wide range of roles peers serve in or the highly variable nature of their professional involvement with this work (e.g., *ad hoc*, lay, peer volunteers vs. full-time, trained, paid peer workers). In many clinical settings, unpaid lay peers are called upon to provide support to patients with SUD across all stages of recovery.

Common functions of PRSS include facilitating and supporting patients' engagement with SUD treatment and transition between levels of care (e.g., between inpatient and outpatient programs), in addition to connecting patients with community based recovery support services and mutual-help organizations in ways not possible for conventional treatment providers who are bound by ethical considerations like not forming dual relationships with patients (Valentine, 2010; White and Evans, 2014). PRSS can also help individuals navigate systems to build recovery capital, attain employment, attend mutual-help groups, and address criminal justice issues.

Probably the largest area of SUD peer-service growth over the past decade, however, has been in the uptake of peer recovery coaches. Recovery coaches are peers trained to provide informational, emotional, social, and practical support services to people with alcohol or other drug problems through a wide variety of organizational sponsors, including recovery community centers, as well as hospital and outpatient clinical settings (White, 2009). Typically they are paid employees working part- or full-time with some degree (a high school diploma or GED is usually required) of formal training and certification. Due to lack of agreed standards in terminology, in some clinical settings the term recovery coach may also refer to “recovery allies” who support individuals with SUD, but do not have lived experience with addiction. Such supports are not covered in this review.

Regardless of the nature of their role, peers have the ability to engage patients outside the confines of traditional clinical practice. This ability to fill critical care gaps is the most probable reason for their widespread uptake across a diverse range of SUD treatment settings and the reason they have emerged as a critical component of recovery management (White, 2009). SAMHSA has made efforts to identify and describe core competencies for peer support workers in working with individuals with SUD as well as other psychological disorders (SAMHSA, 2015), and with time, PRSS roles and qualifications will become better defined.

While a compelling case has been made for PRSS in a number of theoretical articles and book chapters (e.g., White, 2009, 2010, 2011; Bora et al., 2010; Cicchetti, 2010; Valentine, 2010; Powell, 2012; Laudet and Humphreys, 2013; White and Evans, 2014), to date empirical research on the topic is somewhat limited. Previous reviews of the PRSS literature published in Reif et al. (2014) and Bassuk et al. (2016) reported that overall, existing research at the time showed PRSS were commonly associated with reduced substance use and SUD relapse rates, improved relationships with treatment providers and social supports, increased treatment retention, and greater satisfaction with treatment. Bassuk et al. ultimately concluded that there is evidence for the effectiveness of PRSS. Overall, however, both reviews highlighted concerns about the methodological rigor of the then existing research, which included an inability to distinguish the effects of peer recovery support from other recovery support activities, small samples and heterogeneous populations, inconsistency in the definitions of peer workers and recovery coaches, lack of any, or appropriate comparison groups, and inconsistencies in the quantity of peer-provider supervision. Ultimately, Bassuk et al. noted that although evidence for the effectiveness of PRSS exists, these limitations should offer pause, and that additional research is necessary to determine the effectiveness of different peer approaches and types of peer support services, with regard to the amount, intensity, peer skill level, service context, and effectiveness among different populations served.

PRSS, and recovery coaching models are increasingly and rapidly being rolled out in health care settings, despite little empirical knowledge of best practices and sense of to what degree services will help, and for whom. The aim of the present article is, therefore, to report the most up to date research on PRSS through systematic review. This review includes six new articles published following Bassuk et al.'s review. It also extends previous reviews by utilizing broader inclusion criteria (e.g., including cross-sectional studies and clinical interventions linking patients to 12-Step programs using 12-Step program volunteers) that provides broader context for this fast-growing literature. The review also identifies, wherever possible, for whom and under what conditions PRSS may have utility to inform health care and community-based PRSS delivery. We also highlight important gaps in the knowledge base that will inform the direction and scope of treatment and future research in this important, emerging area.

## Methods

A systematic search of the literature (as of 10/13/2018), using the search terms “recovery coaching,” “peer recovery support,” “peer-based recovery support services,” and “individual peer support” in combination with substance use terms, identified 158 records across four publicly available databases (i.e., PubMed, EMBASE, CINAHL, and PsycInfo; see Appendix A in Supplementary Material for search term syntax). Given the relative novelty of this line of investigation we cast a wide net in terms of article inclusion criteria. We included randomized controlled trials (RCTs), quasi-experimental studies, single- and multi-group prospective and retrospective studies, and cross-sectional/descriptive studies related to SUD. All age ranges, substances used, and available outcomes were included. Non-peer reviewed items, however, were not included (e.g., book chapters, dissertations, institutional reports). Reports had to include at least one substance use or related outcome.

**Figure 1:**
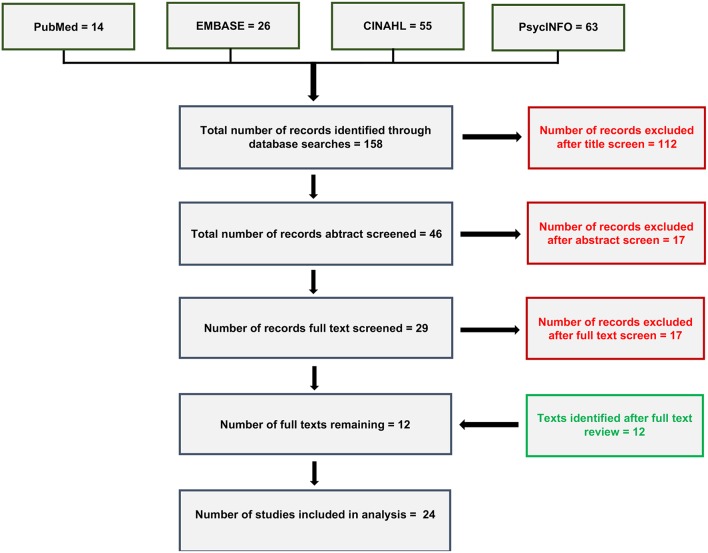
Literature review diagram showing article review and selection.

A title screen removed 101 duplicate records, and 11 records on non-relevant topics (e.g., peer support for recovery for problem unrelated to addiction). An abstract review removed an additional 17 records: seven book chapters (removed because they were not peer reviewed and did not report original data), seven records on non-relevant topics, two review articles, and one article because it reported on a mandated to treatment sample. A full text review removed another 17 records: seven review and ten theoretical articles. The remaining 12 studies were included in the analysis and are summarized in Table S1 (Supplementary Material) in addition to 12 relevant articles identified subsequently (see Figure 1, literature review diagram) resulting in 24 included reports.

## Results

### Results Overview

We found seven RCTs, four quasi-experiments, as well as eight single- or multi-group prospective or retrospective studies, and two cross-sectional investigations conducted on this topic. The review included 24 reports from 23 original studies containing a total of 6,544 participants. On average, the reviewed studies included more men than women (females, 37.3%; males, 62.7%), although in the majority of studies the racial makeup of samples was diverse, and representative of the populations being studied. Outcomes reported were varied and included self-reported and bioassayed substance abstinence vs. non-abstinence, Addiction Severity Index scores (Mclellan et al., 1992), outpatient substance use treatment attendance, 12-Step meeting attendance, general medical, and mental health appointment adherence, utilization of inpatient substance use treatment services, inpatient readmissions, social functioning, number of psychiatric hospitalization nights, length of living in the community without rehospitalization, number of rehospitalizations, criminal charges, and deaths. The range of follow-up length varied from 1 week to 3 years following the intervention. Below we summarize the review findings by study design type from the most to the least, scientifically rigorous design types.

### Randomized Controlled Trials

Bernstein et al. (2005) conducted the first RCT of a peer recovery support intervention in a sample of 1,175 individuals with SUD reporting past 90-day cocaine and/or heroin use who were receiving general medical care from an urban hospital walk-in clinic, but not SUD treatment. Participants engaged in one of two interventions: either a brief, single session, structured peer education session targeting drug use cessation, which included written advice and a referral list as well as a “booster” telephone call (experimental group), or written advice and referral list for treatment only (control group). Compared to controls, at 6-month follow-up participants receiving a brief peer-support intervention were more likely to be abstinent from cocaine, and trended toward greater heroin, and combined cocaine and heroin abstinence (*p* = 0.05), with OR's 1.51–1.57. This favorable abstinence outcome, however, was not supported by bioassay results; no significant between group differences were observed for bioassayed drug use. Similarly, Addiction Severity Index drug subscale and medical severity scores were not significantly different, and no group differences were noted in detoxification or treatment admissions among those who were abstinent. It is possible that a brief, single-session peer interaction is not sufficient to elicit statistically significant levels of behavior change in individuals with SUD. This does not necessarily preclude the possibility that more intensive or sustained peer contact would achieve this end.

In a demographically similar sample, and using a more protracted treatment protocol, Rowe et al. (2007) compared the effectiveness of clinician-delivered “Citizenship Training” (which included twice-weekly 2-h classes over 8 weeks supporting social participation and community integration) + peer support combined with standard clinical treatment (experimental group), with standard clinical treatment alone (control group), for reducing alcohol and other drug use, and number of criminal justice charges (*N* = 228). Participants were adult outpatients with severe mental illness who had criminal charges within the 2 years prior to study enrolment. Though having an SUD was not required for study participation, the majority of study volunteers had either a primary or secondary SUD diagnosis. Over the 4-month study period participants attended an average of 66% of Citizenship Training classes, and met once weekly with their peer-mentor. A significant group × time interaction showed participants randomized to the peer support group showed reduced alcohol use over 6- and 12-month follow-up as measured by the Addiction Severity Index alcohol use subscale (*d's* = −0.22 and −0.43, respectively), while controls demonstrated increased drinking over the same periods. A similar group × time interaction was not reported for drug use measured by the Addiction Severity Index drug use subscale, although from baseline to 6-month follow-up the peer support group showed reduction in drug use (*d* = −0.62), while the Citizenship Training group showed an increase (*d* = 0.27). From baseline to 12-month follow-up, however, both groups showed reductions in drug use, though the effect size of this reduction was notably larger for the group receiving peer support (peer support group *d* = −0.64; Citizenship Training *d* = −0.16). It is not clear, however, whether these effects were driven by the Citizenship training itself, peer support, or a combination of the two. Also, given only 31% of the sample had alcohol use disorder, it is not clear how clinically meaningful this reduction is. Both control and experimental groups demonstrated significantly less non-alcohol drug use and had fewer criminal justice charges over the 12-month study period signaling that on these measures, Citizenship Training + peer support did not perform better than standard clinical treatment alone.

Three RCTs have also been conducted in which peer volunteers from 12-Step groups were brought into the clinical milieu to help connect patients receiving outpatient treatment for SUD to 12-Step programs in the community. Timko et al. (2006) developed and tested a brief, three-session, intensive referral to 12-Step intervention for Department of Veterans Affairs outpatients (*N* = 345). Participants were randomly assigned to a standard referral in which they were given a schedule for local 12-step meetings and were encouraged to attend, or intensive referral to 12-Step that included linking patients to 12-Step volunteers and using journals to check meeting attendance. For those receiving intensive referral, counselors arranged a meeting between the patient and a participating member of a local Alcoholics Anonymous or Narcotics Anonymous group by calling the peer volunteer in-session to arrange for them to meet patients before a 12-Step meeting so that they might attend the meeting together. Intensive referral was associated with greater likelihood of being involved with 12-Step groups and better alcohol and other drug use outcomes over a six-month follow-up period. Subsequently, Timko and Debenedetti (2007) followed up with these participants at 1 year and found the benefits of intensive referral were sustained. The intensive referral group were more likely to attend at least one meeting per week (OR = 1.38), and had greater 12-Step group involvement (*d* = 0.23), as well as high rates of abstinence (OR = 1.61).

Later, Timko et al. (2011) employed a very similar intervention structure, but with a sample of dually-diagnosed individuals seeking outpatient treatment at the Veteran's Administration. Participants were randomized either standard referral, or four sessions of intensive referral to Double Trouble in Recovery—a 12-Step program for individuals with SUD and co-occurring psychiatric conditions. Intensive referral included a peer volunteer from Double Trouble in Recovery joining participants and their counselor in session. Peers gave a brief personal history and arranged to meet participants and attend a meeting together. At 6-month follow-up those receiving intensive referral were more likely to have attended a Double Trouble in Recovery meeting, and had attended more meetings (*d* = 0.89). Similarly, these participants were also more likely to have attended other 12-Step program meetings, and had greater frequency of attendance at these meetings (*d* = 0.25). They also had less past 30-day drug use (*d* = 0.30) and fewer psychiatric symptoms (*d* = 0.28). No differences were observed for alcohol use and notably only 23% of patients in the intensive-referral group actually attended a Double Trouble in Recovery meeting during the 6-month follow-up period compared to 13% in the standard referral group, suggesting about one-fifth of participants receiving intensive referral were driving the observed between group differences.

Manning et al. (2012) sought to determine whether peer referral to 12-Step meetings would increase 12-Step meeting attendance among individuals with SUD undergoing inpatient detoxification (*N* = 151). Patients were randomized to either, (1) introduction and referral to 12-Step by a peer who shared their own recovery experience with the participant, (2) introduction and referral to 12-Step by a doctor, or (3) no introduction or referral (control group). Peers and doctors were instructed to initiate and maintain an open dialogue with participants about their beliefs, concerns, and experiences with 12-Step meetings, and to address any concerns or misconceptions that clients may have held about 12-Step meetings. Together, peer and doctor referral to 12-Step led to increased attendance at 12-Step meetings during inpatient treatment (88 vs. 73%), though peer and doctor groups had similar rates of 12-Step meeting attendance on the inpatient unit (89 and 87%, respectively). Rates of post-discharge meeting attendance, however, were significantly higher in the peer referral group (64%; OR = 3.6) compared to the doctor referral (48%) or no referral groups (33%). Further, participants who attended 12-Step meetings while inpatient were three times as likely to have attended meetings post-discharge than those who did not attend 12-Step meetings while inpatient (59 vs. 20%), and post-discharge meeting attenders reported significantly higher abstinence rates at 3-month follow-up (60.8 vs. 39.2%). Abstinence rates at 3-month follow-up, however, did not differ significantly across intervention groups. Taken together, findings suggest introduction and referral to 12-Step programs for individuals in inpatient detoxification increases 12-Step meeting attendance both during inpatient treatment and after discharge, and that meeting attendance is associated with higher abstinence rates; it is not necessarily important, however, that these referrals/introductions be peer-delivered.

In contrast to the aforementioned studies, which utilized either single session, peer-delivered intervention (Bernstein et al., 2005) or peer support as an addendum to a professional-delivered treatment (Rowe et al., 2007). Tracy et al. (2011) compared a peer-driven treatment that included peer-led groups as well as peer support, to a professional-delivered treatment with peer support in a sample of 96 Veterans Administration inpatients. Study groups included, (1) treatment as usual (TAU) combined with peer-led groups and weekly peer mentorship, (2) TAU combined with a dual recovery intervention involving 8 weeks of clinician-delivered individual and group relapse prevention therapy in addition to peer-led groups and weekly peer mentorship, and (3) TAU only. TAU consisted of standard coping/skills training groups, medication management, and social work support to handle basic needs during inpatient stay. Substance misuse, psychiatric, and medication management support services were also available. Peer mentors were referred by their treating physician/clinician to a compensated work therapy program, and screened by the program coordinator and mentor supervisor from clinical record and interview. 88% of study participants had an alcohol use disorder or other SUD, in addition to psychiatric comorbidity. TAU combined with peer-delivered treatment, and TAU combined with professional-delivered treatment and peer support were both associated with greater post-discharge, outpatient substance use treatment attendance compared to TAU alone (51 and 52% SUD treatment appointment adherence respectively among those receiving peer ministrations vs. 38% for TAU). These two interventions were also associated with greater general medical, and mental health appointment adherence (43 and 48% appointment adherence respectively among those receiving peer ministrations vs. 33% for TAU), as well as greater inpatient substance use treatment accessed (*d's* = 0.33 and 0.63 respectively vs. TAU only). Taken together, findings suggest that at least in terms of treatment adherence, compared with TAU alone, interventions including peer support or peer delivered ministrations are superior. Substance use outcomes were not reported.

Most recently, O'Connell et al. (2017) recruited 137 inpatients with psychotic disorders and co-occurring problematic substance use through substance dependence to receive either, (1) TAU with skills training, (2) TAU with skills training + the “Engage Program,” which included contact with a peer support while inpatient, peer home visits after discharge, twice-weekly mutual support groups accompanied by the peer, and social and recreational outings, or (3) TAU only (not defined by the study's authors). Interventions were begun while participants were on an inpatient unit, and continued for 3 months post-discharge. At 3-month follow-up, participants receiving TAU with skills training, and TAU with skills training + the “Engage Program” fared better than those receiving TAU only in terms of reduced alcohol use (*d's* = −0.54 and −0.81 respectively vs. TAU only), and alcohol use disorder symptom endorsement (*d's* = −1.23 and −1.47 respectively vs. TAU only). Those in the Engage Program also viewed getting help for their alcohol use problems as being more important compared to those receiving TAU only (*d* = 0.69), though differences between those receiving peer support and those receiving TAU with skills training were not significantly different. Notably, Participants in the Engage group had significantly greater increases in self-criticism from baseline to 3 months compared to those receiving TAU (*d* = 0.43), which the authors posit may be a function of peer staff holding up higher expectations for their clients than clinical staff. Additionally, 6 months into the study, participants in the Engage Program had greater duration of outpatient service use compared to those in the TAU group (*d* = 0.31). At 9-month follow-up, skills training, and skills training with peer support was associated with fewer positive psychotic symptoms and greater functioning in comparison to TAU only, suggesting no specific effect of peers on these measures at this measurement time point. Participants in the peer support and skills training only groups also had significantly fewer psychiatric hospital readmissions from baseline at 6 and 12 months compared to the TAU group, though the peer support and skills training only groups were not significantly different from one another on this measure.

#### Summary of Randomized Controlled Trial Evidence

Taken together, the RCTs reviewed here had a number of strengths, including strong research designs, provision of manualized treatment for the clinical components of studies (Bernstein et al., 2005; Timko et al., 2006, 2011; Tracy et al., 2011; O'Connell et al., 2017), and samples with diversity in terms of sex and race. Notable limitations, however, include generally poorly defined and non-manualized peer roles and procedures, although some studies incorporated semi-structured scripts (Bernstein et al., 2005) or manualized training protocols (Tracy et al., 2011) for their peer workers, and combining of peer services with clinician-delivered interventions without the necessary control groups to allow discernment of the independent effects of peers (Rowe et al., 2007; Tracy et al., 2011). Overall, positive effects appeared small to moderate in magnitude, and null findings were observed for many hypothesized treatment effects. It's possible too that the large numbers of measures assessed across these studies could be leading to type I error. These findings, however, should be taken in context; these studies typically reported on novel interventions still under development, providing treatment for individuals with complex clinical presentations (i.e., co-occurring mental disorders in addition to SUD), high addiction severity, and significant SUD related challenges such as homelessness.

### Quasi-Experimental Studies

Quasi-experimental studies addressing PRSS generally align with findings from the aforementioned RCTs. In an early study investigating the potential of PRSS, Sisson and Mallams (1981) sought to increase the likelihood of participation in Alcoholics Anonymous and Al-Anon meetings among a sample of adults receiving outpatient treatment for alcohol use disorder (*n* = 16) and their spouses (*n* = 4) in a sparsely populated, rural area. Participants were randomly assigned to either a standard referral procedure which involved receiving information about Alcoholics Anonymous or Al-Anon, and providing information concerning time, date, and location of weekly meetings with encouragement to attend (control group), or to systematic encouragement and connection to 12-Step groups that involved a phone call being made in a counseling session to an Alcoholics Anonymous or Al-Anon member, who had volunteered to provide peer support. The 12-Step group member briefly talked to participants about 12-Step meetings, offered to give a ride to a meeting or meet them before a meeting, and followed up with a call the night of the meeting to remind them about it and to encourage them to attend (experimental group). One hundred percent of the experimental group attended an Alcoholics Anonymous or Al-Anon meeting within 1 week of referral and continued to attend, whereas none of the control group attended a meeting. The mean attendance rate over four-week follow-up was 2.3 meetings for the experimental group and zero for controls, and (*d* = 2.74). It is possible that peer linkage helped individuals surmount barriers to attending initial 12-Step meetings due to factors like distance needed to travel to meetings such rural areas.

In a similar study with a sample of patients hospitalized for alcohol and other drug detoxification, Blondell et al. (2008) utilized 12-Step group volunteers to visit patients undergoing medical detoxification (*n* = 19). During visits, which would typically last between 30 and 60 min, peers would explain how involvement in mutual-help programs was an essential part of their recovery from SUD. The control group (*n* = 80) consisted of usual care in which mutual-help meetings were available every evening, but attendance was not required. The authors found that the brief, single-session peer-delivered counseling intervention resulted in greater likelihood of completion of medical detoxification and not leaving “against medical advice” (88% completion vs. 74%). Although peer visits did not result in statistically significant differences in mutual-help meeting attendance following detoxification (*p* = 0.05), observed differences were clinically meaningful (90% attendance for those receiving peer visits vs. 64% for those not). Similarly, likelihood of abstinence from all substances seven days after discharge was 84% for those receiving peer visits vs. 59% for those not (*p* = 0.06), and initiation of professional aftercare treatment at 1-week follow-up post detoxification discharge was 100% for those receiving peer visits vs. 82% for those not (*p* = 0.06). While many detoxification sites invite 12-Step groups to bring meetings into units, this work suggests the possibility of added benefit to allowing 12-Step group members to meet individually with patients to share their experience of recovery, and encourage and support meeting attendance.

Work by Boisvert et al. (2008) indicates that PRSS may also bolster patients' perceived support. Using a sequential cohort comparative design and a sample of adults with SUD and severe mental illness living in permanent supportive housing (*N* = 19), the authors found that 10 individuals who participated in a peer-driven program based on recovery community model published by SAMHSA and did not relapse, reported increased perceived emotional/informational (*R*^2^ = 0.39), tangible (*R*^2^ = 0.24) and affectionate support (*R*^2^ = 0.24) from pre- to post-intervention. Additionally, participants receiving the peer-support recovery program had lower rates of return to homelessness (85 vs. 33%) over a 6-month period, compared to a sample of residents living in the permanent supportive housing setting 6-months prior to instigation of the peer-support program. Further, prior to institution of the peer program, residents had a 24% chance of relapse to substance use, while the risk for those residents participating in the program was 7%, though it is not clear if this difference was statistically significant and no demographic or clinical data were provided for this comparison group.

Working in the Veteran's Administration system, Smelson et al. (2013) assessed a novel program referred to as Maintaining Independence and Sobriety Through Systems Integration, Outreach, and Networking (MISSION) for military veterans with SUD and co-occurring mental disorders, as well as experienced homelessness and current unemployment using a quasi-experimental, intact group design (*N* = 333). Over 12 months, MISSION provides temporary housing, and delivers integrated mental health and SUD treatment delivered via Dual Recovery Therapy (Ziedonis and Stern, 2001), case management, and vocational and peer support. The manualized program is delivered by a case manager and peer specialist team. Those receiving MISSION had greater outpatient session attendance within the 30 days before the 12-month follow up (*d* = 1.25), and a greater decline in the number of psychiatric hospitalization nights compared to those receiving TAU only (*d* = −0.26). Both groups, however, showed improvement on measures of substance use and associated problems at 12 months, though those receiving MISSION were less likely to drink to intoxication (OR = 0.29) and experience serious tension or anxiety (OR = 0.53). Given the broad treatment platform in this study, it is impossible to separate out peer effects. The findings nevertheless speak to the promise of integrating peer supports with clinician-delivered treatments.

Most recently, in a large sample of parents or caregivers referred by child protective services to a specialized SUD outpatient treatment program (*N* = 1,362), James et al. (2014) found that peer contact was associated with faster outreach, and shorter latency to initial clinical assessment (*d* = 0.16), as well as higher rates of any treatment service initiation compared to no peer contact (96.9 vs. 89.9%). However, when the authors used a more restrictive definition of service initiation—limited to initiation of individual, group, or family counseling, 84.88 and 82.53% of individuals referred to the enhanced and standard programs, respectively, initiated these services. Those receiving PRSS were less likely to complete treatment (26.64 vs. 38.12%), however, among those completing treatment, the average length of treatment was significantly greater for the PRSS + TAU group than controls (*d* = 0.35). Additionally, participants who had received PRSS who discontinued treatment remained in treatment longer than controls who discontinued treatment (*d* = 0.36). Groups, however, were not significantly different in terms of total numbers making it to initial assessment appointments, initiating counseling, or discontinuing participation in treatment. Notably, relative treatment dropout rates were very high for both the PRSS (56.9%) and control groups (52.9%), though the difference was not statistically significant (*p* > 0.05). Also, effect sizes were generally small suggesting the large sample size may have been driving observed statistically significant effects.

#### Summary of Quasi-Experimental Evidence

Quasi-experimental studies to date provide further support for the potential of PRSS for SUD. The quasi-experimental literature, however, includes many of the limitations observed for the RCT literature. For instance, peer roles were typically not well defined, nor were peer training protocols well-articulated. Further, positive findings were often small to moderate in size and no studies included intent-to-treat design meaning participants who dropped out of interventions or relapsed were not included in many of the analyses. Although it is difficult to parse out the independent effect of peers—because with the exception of Sisson and Mallams (1981) and James et al. (2014) these studies lacked the necessary control groups—overall these findings suggest PRSS may have the ability to sure up treatment attendance and help individuals engage with treatment. These findings also speak to the versatility of PRSS by showing a diverse range of residential treatment settings in which peer services might be utilized.

### Single- or Multi-Group Prospective or Retrospective Studies

Single- or multi-group prospective or retrospective studies addressing PRSS extend the case for more research on PRSS. Boyd et al. (2005) piloted a 12-week peer-delivered psychoeducation program for women with HIV living in rural areas. Though no inferential analyses were conducted due to the small sample size (*N* = 13), results intimate the authors' brief peer-counseling intervention may increase participants' recognition that their alcohol and other drug use is problematic, and increase the likelihood of steps being taken to address their alcohol and other drug use. The authors highlight the difficulty in identifying and retaining peer counselors for a majority of the rural U.S. areas where this pilot study was implemented, speaking to some of the real-world challenges associated with implementation of PRSS, especially in already underserved geographic areas. This observation speaks to the potential utility of peer coaching via telemedicine (Huskamp et al., 2018).

Using government public health, and Medicaid records, Min et al. (2007) retrospectively assessed whether a long-term, peer-mentorship intervention for individuals with SUD and severe co-occurring mental illness has the capacity to reduce rehospitalization rates (*N* = 484). Survival analysis results over a 3-year period indicate that peer-support program participants had longer periods living in the community without rehospitalization, and a lower overall number of rehospitalizations, compared to a sample of comparable controls not engaged in peer-mentorship.

Similarly, Andreas et al. (2010) shared preliminary findings for the Peers Reach Out Supporting Peers to Embrace Recovery (PROSPER) program, which includes peer-run groups, coaching, workshops and seminars, social and recreational activities, and community events (*N* = 509). Peers work closely with program staff and receive extensive training and supervision. Study participants included women and men over the age of 18 who had SUD and histories of incarceration. From baseline to 12-month assessment the authors observed increases in self-efficacy, perceived social support, and quality of life, as well as decreases in perceived stress, though guilt- and shame-based emotions increased over the same period of time.

Work by Armitage et al. (2010) suggests PRSS may also be beneficial to individuals in sustained SUD remission. The Recovery Association Project (RAP), which emphasizes active citizenship and social engagement, is facilitated by individuals in recovery from SUD who had completed at least 15 h each of RAP leadership training (*N* = 152). The authors found retrospectively that 6 months following RAP participation, 86% of their clients reported no past 30-day alcohol or other drugs use, and another 4% indicated reduced use. Further, 95% reported strong willingness to recommend the program to others, 89% found services helpful, and 92% found provided materials helpful.

Using a multi-group prospective design, Deering et al. (2011) sought to better understand the effects of a peer-led, mobile outreach program for female sex workers. Women were surveyed every 6 months over 18 months (*N* = 242). Women were more likely to utilize the peer-led outreach service if they were at higher risk due to factors such as seeing >10 clients per week, working in isolated settings, injecting cocaine, or injecting/smoking methamphetamine in past 6 months. Utilizers of the peer-led service, however, were also more likely to access the intervention's drop-in center, and notably, after statistically controlling for inter-individual differences, past 6-month use of the peer-led outreach program was associated with a 4-fold increase in the likelihood of participants utilizing detoxification and/or inpatient SUD treatment.

In a retrospective single group study, Kelley et al. (2017) explored the effects of the Transitional Recovery and Culture Program, a Montana-based, community-driven, PRSS intervention aimed at improving sobriety rates in a collection of Native American communities in the region, and increasing community awareness of substance use problems and the need to support SUD recovery (*N* = 224). The authors found that participants completing 6-month follow-up (29%) had significant reductions in past 30-day alcohol (*d* = −0.78) and other drug use (*d* = −0.64). Participants were also more likely to have attained housing and employment. Symptoms of anxiety and depression, however, were not significantly changed. The low follow-up rate (29%) for this study, however, suggests the possibly of selection bias; i.e., individuals lost to follow-up were doing worse and are not represented in the results, making intervention look better than it actually was. As such, these results should be interpreted with caution.

Most recently, Scott et al. (2018) piloted an intervention designed to help link individuals actively using opioids to detoxification and/or agonist medication treatment. Peers approached individuals in urban areas identified as high-risk for continued opioid use and overdose, engaged them in a conversation about heroin, and explained they were recruiting for a study that aimed to help people get into treatment. If the individual expressed interest in the study, the peer outreach worker then called study staff to phone-screened the prospective participant for study eligibility. At the study office, participants met with a treatment linkage manager who used an adapted version of the Recovery Management Checkup protocol (Scott and Dennis, 2010) to link individuals to detoxification and/or methadone agonist medication therapy. Over the course of 8 weeks, peer outreach workers identified 88 individuals actively engaged in opioid use. Seventy-two were screened as eligible, and 70 showed to the treatment linkage meeting. Of those showing up to the treatment linkage meeting, eight went to detox, and nearly all (96%) were admitted to methadone treatment, with a median time from initial linkage meeting to treatment admission of 2.6 days. The majority of participants were still in treatment at 30 and 60 days post-intake (69 and 70%, respectively). This study demonstrates the synergistic potential of integrating peer-based approaches and evidence-based SUD interventions. While peers were not necessarily providing treatment *per se*, they served in this instance, as a critical link to treatment and were able to accomplish in the field what may be difficult for a non-peer provider.

Also interested in the benefits peers can confer for individuals with opioid use disorder, Samuels et al. (2018) explored if connecting individuals presenting to emergency department (ED) for opioid overdose would benefit from PRSS provided in the ED, in addition to provision of naloxone, and usual care consisting of medical stabilization and provision of a list of SUD treatment programs in printed discharge instructions (*N* = 151). Using ED electronic medical record review, they contrasted this intervention to provision of naloxone with written and video instructions on use + usual care, and usual care only. Peers were employed by the partner community-based peer recovery organization. Participants were assigned to one of the three treatment groups based on provider and patient discretion. Peers met with participants in the ED and assessed their readiness to seek treatment, identified overdose risk factors, and provided individualized support and addiction treatment navigation, including linkage to medication for opioid use disorder at the time of, and at least 90 days after the ED visit. The authors did not find significant differences between groups at 12-month follow-up via electronic medical record review; groups were similar in terms of proportion of participants initiating medication for opioid use disorder, number of times returning to the same emergency department for overdose, number of deaths, and median time to death.

#### Summary of Single- or Multi-Group Prospective or Retrospective Study Evidence

While the majority of these single- or multi-group prospective or retrospective studies speak to the promise of PRSS, they should be considered in the light of significant methodological limitations associated with these research designs. Single-group prospective and retrospective designs lack control groups; it is therefore not possible to know if some of the positive findings presented here reflected natural improvements in psychosocial functioning commonly observed in SUD interventions. Relatedly, in multi-group prospective and retrospective studies where comparison groups are used, groups are not selected by random assignment. As such there is risk for selection bias, although the majority of studies reported here checked for demographic between-group differences in order to mitigate this risk. Risk for selection bias is further increased because these studies did not use intent-to-treat analysis; it is thus possible that the benefits conferred by these programs are inflated. Further, all peer-based programs reported here included a wide range of activities and types of support. It is therefore not possible to parse out the unique effects of peers in the context of these interventions.

### Cross-Sectional Investigations

The cross-sectional literature tentatively speaks to the potential of PRSS-based interventions in a range of treatment settings. Sanders et al. (1998) sought to contrast client satisfaction with peer-delivered SUD counseling, and counseling from traditionally-trained addiction counselors (*N* = 56). They found that although there were no between-group differences in overall treatment satisfaction, women receiving ongoing SUD counseling from a peer-counselor were more likely to describe their counselors as empathic, to identify them as the most helpful aspect of the program, to utilize other clinic resources, and to more strongly recommend the treatment program, compared to clients receiving counseling from traditional providers. This work speaks to the ability of peers to establish rapport in patients. It does not however speak to quality of care or treatment outcomes. It is unclear whether professional-delivered treatment may benefit them more in terms of treatment outcomes, even though patients may feel greater affinity for peer counselors.

One study has also assessed the motivation of individuals in recovery from SUD to seek PRSS. Wanting to know more about university students participating in peer-based college recovery support services, Laudet et al. (2016) surveyed 486 students engaged in 29 college recovery programs across the United States. At the time of survey, students had been abstinent from alcohol and other drugs a mean of 3 years. One third of the sample reported they would not be in college were it not for a peer-based, collegiate recovery program, and 20% would not be attending their current university. Top reasons cited for joining collegiate recovery programs were the need for same age peer recovery support, and wanting to maintain their sobriety in the high-risk college environment.

## Discussion

Although a strong theoretical case has been made for the potential utility of PRSS in a range of SUD clinical and care settings (e.g., White and Evans, 2014; Laudet et al., 2016), to date PRSS research is limited for specific clinical SUD populations for whom these services are most commonly provided (i.e., those in outpatient, residential and transitional care settings, and recovery community centers). In their 2016 review of the PRSS literature, Bassuk et al. noted open questions about the necessary amount and intensity of PRSS interventions, and the optimal contexts for provision of these services and the appropriate skill levels for peers. Several years later, though a number of recent studies have begun to inform these considerations, these remain open questions. Moreover, additional work is needed to parse out for whom and under what conditions these PRSS interventions have most utility, and to determine how peers should be trained, and what, if any certifications should be required for peer work in order to inform the development of “best practice” models. Further, research into potential cost-benefits to healthcare systems is necessary. Although the existing literature reviewed here reports mixed findings, positive findings to date speak to the possibility of benefits associated with adoption and implementation of PRSS. When placed in the context of other research in the recovery supports arena (e.g., Humphreys and Moos, 2001, 2007), such entities hold promise as cost-effective care models that can bridge gaps not covered by traditional care.

In theory, peer supports such as recovery coaches may have particular utility in hospital and clinical outpatient settings since many individuals with SUD who are not yet engaged in treatment present to these sites with SUD-related medical problems. Peers are uniquely positioned to engage such individuals and help connect them with SUD treatment, either in hospital systems, or the community. Bernstein et al. (2005) showed that even a single-session peer-led intervention for individuals presenting to a hospital-based, walk-in clinic could result in significant reductions in substance use at a 6-month follow-up. Though this work is promising, more research is needed to determine how effective peer-driven interventions may be. Hospital and medical settings that have begun to utilize SUD peer supports should be encouraged to monitor their programs and where possible report their outcomes.

PRSS may be especially beneficial in substance detoxification units, since successfully connecting individuals to care following detoxification is a persistent and vexing problem for providers. PRSS might also impact the culture of detoxification units by offering a multiple pathways to recovery approach. Blondell et al. (2008) found that detoxification patients receiving a single peer counseling session were more likely to complete medical detoxification and not leave detoxification “against medical advice.” Though differences between participants receiving a peer counseling session and controls were not statistically significant on measures of attendance of mutual-help group meetings during the first week following detoxification discharge, remaining abstinent following discharge, and initiating professional aftercare treatment, statistical trends with clinically meaningful differences were observed suggesting those receiving peer counseling fared better in a detoxification setting already strongly encouraging 12-Step participation. These observed trends may have been statistically significant were the study better powered. Based on these findings, more work in this area is justified. Peer supports could ultimately be a cost-effective way to bridge the gap between detoxification and longer-term SUD treatment by helping patients enter residential programs, and/or engage with recovery programs in the community such as mutual-help groups like Alcoholics Anonymous, Narcotics Anonymous, Refuge Recovery, Rational Recovery, and/or SMART Recovery.

The evidence reviewed here also suggests peer supports may have the ability to improve outcomes for individuals engaged in inpatient or outpatient psychiatric treatment for SUD and co-occurring mental disorders. In such contexts peer supports have been shown to reduce substance use (Rowe et al., 2007; O'Connell et al., 2017), lead to better SUD and medical treatment adherence (Tracy et al., 2011), get individuals to SUD treatment faster following SUD treatment referral (James et al., 2014), reduce the frequency of inpatient readmission (O'Connell et al., 2017), and reduce criminal behavior recidivism (Rowe et al., 2007). This body of work, however, reports a wide range of PRSS outcomes, for which there are also many negative findings showing treatment as usual performed equally well as PRSS interventions. More work is needed to determine the ways peer supports can be most effective in these treatment contexts, and how, in the future, PRSS' efforts might be best focused.

Presently in the Unites States, state-to-state regulations vary greatly in terms of training and credentialing requirements for peer workers (London et al., 2018). More work is needed to determine how peers should be trained, and what, if any certifications should be required for peer work. Studies reporting training procedures utilized a highly variable range of training protocols for peers. Most of these studies report providing some sort of supervision provided by licensed clinicians, though the quantity and frequency of supervision was typically not described. Future research will benefit from more clearly articulating peer roles in published manuscripts (Jack et al., 2018), and where possible, manualizing aspects of peer interventions. This will help future studies replicate findings, and also help educators and treatment providers develop better training protocols for peer workers. Work is also needed that identifies which peer roles are most helpful/effective in different clinical, treatment, and recovery support contexts. Further, it is important that future research distinguishes between paid peer workers such as recovery coaches who are generally expected to have formal training and certification (e.g., Tracy et al., 2011; O'Connell et al., 2017), and untrained, volunteer peer supports who may facilitate brief interventions akin to 12-step calls made by members of mutual-help groups (e.g., Sisson and Mallams, 1981; Blondell et al., 2008).

Community-based SUD programs also utilize PRSS. Research summarized in this review suggests peer recovery supports integrated into community outreach programs may increase individuals' self-awareness of problematic substance use (Boyd et al., 2005), and lead to reductions in alcohol and other drug use (Kelley et al., 2017). Such programs may also lead to greater utilization of detoxification programs and residential SUD treatment among those needing treatment (Deering et al., 2011), and reduce rehospitalization rates following treatment (Min et al., 2007). Findings from these preliminary cross-sectional, and prospective and retrospective studies indicate more comprehensive RCTs are warranted on this topic, and suggest that marginalized and/or stigmatized populations may particularly benefit from peer-driven initiatives.

Relatedly, peers may also have potential to bolster harm reduction programs. Ashford et al. (2018), for instance, found peers could be successfully utilized to engage individuals who are at risk of diseases such as hepatitis-C and HIV, and overdose in the context of an urban needle exchange program. In light of the current opioid crisis, such ministrations are much needed and could enhance existing efforts to curb the prodigious disease burden of opioid misuse.

### Assessment of Potential Bias

The findings reviewed in the present paper should be tempered by the fact the discussed RCTs did not use an intent-to-treat design, potentially introducing sample bias into the results. Additionally, to date, all RCTs studying PRSS have recruited participants with fairly severe SUD and co-occurring mental illness, and major impairment in psychosocial functioning. It is therefore not clear how these results might generalize to samples of individuals with less severe SUD presentations, and those without psychiatric comorbidity. The vast majority of SUD treatment in the US is level-I outpatient treatment, yet to our knowledge there are no studies that have examined the utility of providing peer supports/recovery coaches in these settings. It should also be highlighted that, by nature, much of the non-RCT research presented here is based on convenience sampling, and survey analysis. More RCTs are needed on this topic to validate, and expand upon reported findings.

## Conclusions

This comprehensive, systematic review of the existing PRSS literature speaks to both the potential of peer supports across a number of SUD treatment settings, as well as the great amount of work yet needed to establish the efficacy and effectiveness of such ministrations. Importantly, many ethical and practical challenges remain for this novel class of interventions for SUD. For instance, individuals providing peer support face boundary issues as their work typically lies at the intersection of purely-peer, and purely-clinical support roles (Jack et al., 2018). Their work lacks the clarity of the professional treatment realm with its clear roles, work schedules, and expectations, and marked differentiation between paid professional staff and clients, as well as the mutual-help 12-Step tradition with its own well-articulated, and long-standing peer-support traditions. Regardless, work to date makes the case for further exploration PRSS in a range of SUD-related contexts. Peer support specialists' roles will, no doubt, increasingly become more clearly defined as peer-supports are integrated more and more into the spectrum of SUD care.

## Author Contributions

JK, BH, and BB conceived the project. LH, CV, and AA conducted literature search. DE and JK wrote manuscript. LH, CV, AA, BB, BH, and CW provided revisions and edited the manuscript.

### Conflict of Interest Statement

The authors declare that the research was conducted in the absence of any commercial or financial relationships that could be construed as a potential conflict of interest.
