# Supplementary material for: Lived Experience in New Models of Care for Substance Use Disorder: A Systematic Review of Peer Recovery Support Services and Recovery Coaching
Source: Front Psychol. 2019 Jun 13;10:1052. doi: 10.3389/fpsyg.2019.01052 (PMC6585590; doi:10.3389/fpsyg.2019.01052)
Supplement: Supplementary file 1 [file Table_1.pdf]

Table 1. Tabularized summary of the evidence on peer-based recovery support services (PRSS)

Article	Study design	Intervention(s)	Description of sample & peers	Sample size (*N*)	Follow-ups	Retention rate	Primary substance	Substance use and related outcomes
Bernstein et al., 2005	Randomized controlled trial	Exp: A single, structured encounter targeting cessation of drug use, conducted by peer educators in the context of a routine medical visit.<br>Con: Written advice only.	Sample: Out of treatment adults with past 90-day cocaine and/or heroin use attending a hospital walk-in clinic.<br>Peers: Experienced substance use outreach workers; level of training not described.	*N* = 1,175<br>(F= 29%, M= 71%)	3 and 6 months	66%	Multi-substance	Compared to controls, at 6-month follow-up, participants receiving a brief peer-support intervention were more likely to be abstinent from cocaine, and trended toward greater heroin, and both cocaine and heroin abstinence (p= .05; OR's 1.51 – 1.57). A trend was also observed in bioassay measured cocaine use, but not heroin use. No group differences were noted in detoxification or treatment admissions among those who were abstinent. Those receiving the peer-support intervention demonstrated a trend toward greater reductions in Addiction Severity Index drug subscale and medical severity scores (p= .06).
Timko et al., 2006 & 2007	Randomized controlled trial	Exp: Intensive referral to 12-Step in which participants were given AA or NA meeting schedules from counselors in addition to information about	Sample: Patients entering SUD out-patient treatment at a Department of Veterans Affairs program.	*N* = 345<br>(F= 2%, M= 98%)	6 months	81%	Multi-substance	Among patients with relatively less previous 12-Step meeting attendance, intensive referral was associated with more meeting attendance during follow-up than was

	12-Step philosophy and the structure and terminology of 12-Step groups over a minimum of 3 sessions in 1 month. Common concerns were addressed, and participants were encouraged to set goals for attending meetings, working the first steps, joining a home group and getting a sponsor. The counselor and patient also called a 12-Step volunteer during session 1 and the volunteer arranged to meet the patient before an AA or NA meeting so that they could attend the meeting together. Participants also received relapse prevention training and psychoeducation about substance misuse consequences, and healthy living.<br><br>Con: Standard referral to 12-Step in which participants were given AA or NA meeting schedules from counselors + relapse prevention training and	Peers: Alcoholics Anonymous and Narcotics Anonymous members who were untrained and unpaid, volunteering support in the context of 12th step work.			standard referral. Compared with those randomized to standard referral, those randomized to intensive referral were more likely to be involved with 12-Step groups during the 6-month follow-up period. Intensive referral patients also had better alcohol and drug use outcomes at 6 months. 12-Step involvement mediated part of the association between referral group and alcohol outcomes.<br><br>At 1-year follow-up (Timko and DeBenedetti, 2007), participants receiving intensive referral were more likely over the past year have attended at least one meeting per week (OR= 1.38), and had greater 12-Step group involvement (*d*= 0.23) and abstinence rates (OR= 1.61). 12-Step involvement mediated the association between referral group and alcohol and drug outcomes, and was associated with better outcomes above and beyond group attendance.

		psychoeducation about substance misuse consequences, and healthy living over a minimum of 3 sessions in 1 month.						
Rowe et al., 2007	Randomized controlled trial	Exp: A community-oriented group intervention with ‘Citizenship Training’ and peer support combined with standard clinical treatment, including jail diversion services.<br>Con: Standard clinical treatment with jail diversion services only.	Sample: Adult outpatients with severe mental illness who had criminal charges within the two years prior to study enrolment, 31% with alcohol use disorder, 42% with other SUD.<br>Peers: Six peer mentors were utilized; all were diagnosed with a serious mental illness and were in treatment. All completed a training program covering confidentiality, the client engagement process, cultural competence, and the distinctive roles of criminal justice and mental health treatment system workers.	*N* = 114<br>(F= 32%, M= 68%)	6 and 12 months	61%	Multi-substance	Four months of ‘Citizenship Training’ geared toward social participation and community integration + peer mentorship, and standard clinical treatment including jail diversion services, produced reduced alcohol use over 12-month follow-up (*d*= –0.43), while those receiving standard clinical treatment with jail diversion services alone demonstrated increased drinking over the same period. Both groups demonstrated significantly less non-alcohol drug use and fewer criminal justice charges over the 12-month follow-up period (peer support group *d*= –0.64; Citizenship Training *d*= –0.16).
Timko et al., 2011	Randomized controlled trial	Exp: Intensive referral to the Double Trouble in Recovery 12-Step program including a counselor-delivered	Sample: Dually-diagnosed individuals seeking outpatient treatment at the Veteran’s Administration.	*N* = 287<br>(F= 9%, M= 91%)	6 months	80%	Multi-substance	Participants in the intensive referral group were more likely to attend and be involved Double Trouble in Recovery (*d*= 0.89) as

		introduction to the program plus information about the its philosophy, structure, and terminology over 4 sessions in 1 month. A volunteer member of Double Trouble in Recovery joined participants and counselors in a session during which the volunteer gave a brief personal history and arranged to attend a meeting with patients.<br><br>Con: Standard referral to Double Trouble in Recovery in which participants were given meeting schedules by counselors and encouragement to attend.	Peers: Double Trouble in Recovery members who were untrained and unpaid, volunteering support in the context of 12th step work.					well as other 12-Step programs (*d*= 0.25), and had less drug use (*d*= 0.30) and fewer psychiatric symptoms (*d*= 0.28) at 6-month follow-up. However, only 23% of participants in the intensive-referral group attended a DFG meeting during the six-month follow-up period compared to 13% in the standard referral group.
Tracy et al., 2011	Randomized controlled trial	Exp: 1) Mentorship for Addictions Problems to Enhance Engagement to Treatment (MAP-Engage): A peer-driven intervention with open-ended individual peer contact and peer-led groups. Peers escort patients to first outpatient program.<br><br>2) Dual Recovery	Sample: Adult inpatients at Veteran's Administration with high hospitalization recidivism and current and/or past diagnosis of SUD, and two or more past-year hospitalizations. 88% had current alcohol or other SUD in addition to	*N* = 96<br><br>(F= 3%, M= 97%)	12 months	100%	Multi-substance	Compared with TAU alone, MAP-Engage, and MAP-Engage + Dual Recovery Treatment were both associated with greater post-discharge, outpatient substance use treatment attendance, general medical, and mental health services appointment adherence, and greater utilization of inpatient substance use treatment services (*d's*=

		Treatment + MAP-Engage: Dual Recovery Treatment is an intervention involving 8 weeks of clinician-delivered individual and group relapse prevention therapy.<br><br>Con: TAU only, consisting of standard coping/skills training groups, medication management, and social work support to handle basic needs during inpatient stay. Substance misuse, psychiatric, and medication management in addition to social work services were also made available.	psychiatric comorbidity.<br><br>Peers: Compensated through work therapy program, and screened by the program coordinator and mentor supervisor from clinical record and interview. Peer mentors were supervised by clinicians, though their level of formal training was not described.					0.33 and 0.63 respectively versus TAU only).
Manning et al., 2012	Randomized controlled trial	Exp: 1) Peer referral to 12-Step meetings.<br><br>2) Doctor referral to 12-Step meetings.<br><br>Con: No introduction or referral.	Sample: Individuals with SUD undergoing inpatient medical detoxification.<br><br>Peers: Alcoholics Anonymous, Narcotics Anonymous, or Cocaine Anonymous members with at least three years of recovery.	*N* = 151<br><br>(F= 33%, M= 67%)	3 months	83%	Multi-substance	Both peer and doctor referral to 12-Step programs increased attendance at 12-Step meetings during inpatient treatment. Rates of post-discharge meeting attendance were greatest in the peer-referred group (OR= 3.6). Inpatient meeting attenders were 3 times as likely to have attended 12-Step meetings post-discharge, and post-discharge meeting

								attenders reported significantly higher abstinence rates at 3-month follow-up. Follow-up abstinence rates did not differ significantly across intervention groups.
O'Connell et al., 2017	Randomized controlled trial	Exp: 1) TAU + a manualized skills training intervention for persons with co-occurring disorders in addition to peer-led social engagement program.<br>2) TAU + a manualized skills training intervention for persons with co-occurring disorders.<br>Con: TAU only, not defined by the study's authors.	Sample: Individuals with co-occurring psychosis and substance use or dependence were recruited during an inpatient psychiatric hospitalization.<br>Peers: Individuals in recovery trained to provide peer support.	*N* = 137<br>(F= 34%, M= 66%)	3 and 9 months	47%	Alcohol	At 3 months, TAU + skills training with and without peer support were effective in reducing alcohol use (*d's*= –0.81 and–0.54 versus TAU only) and related symptoms (*d's*= –1.47 and–1.23 versus TAU only), with the addition of peer-led support resulting in higher levels of relatedness, self-criticism, and outpatient service use. At nine months, skills training was effective in decreasing symptoms and inpatient readmissions and increasing functioning, with the addition of peer support resulting in reduced alcohol use.
Sisson & Mallams, 1981	Quasi-experiment	Exp: Systematic encouragement and community access procedure involving a phone call being made in a counseling session to a local Alcoholics Anonymous or Al-	Sample: Patients receiving outpatient treatment for alcohol us disorder.<br>Peers: Alcoholics Anonymous and Al-Anon members who were untrained and unpaid, volunteering	*N* = 20<br>(F= 30%, M= 70%)	4 weeks	100%	Alcohol	100% of the experimental group attended AA or Al-Anon within 1 week of referral and continued to attend with an average of 2.3 meetings attended over 4-week follow-up, whereas none of the

		Anon member in which the member briefly talked to participants about meetings, offered to give them a ride to a meeting or meet before a meeting. The AA or Al-Anon member then called the participant the night of the meeting to remind them about it and to encourage them to attend.<br><br>Con: Standard referral procedure which involved giving information about AA or Al-Anon, encouraging meeting attendance, and providing information concerning time, date, and location of weekly meetings.	support in the context of 12th step work.					control group ever attended (*d*= 2.74).
Blondell et al., 2008	Quasi-experiment	Exp: A single, 30-60 minute session in which peers in SUD recovery share their personal experience with patients to provide emotional support, enhance motivation to maintain abstinence, and encourage the patient to attend inpatient treatment and/or mutual-help support group	Sample: Patients, hospitalized for alcohol and other drug detoxification.<br><br>Peers: 12-Step program members who were untrained and unpaid, volunteering support in the context of 12th step work.	*N* = 119<br><br>(F= 25%, M= 75%)	1 week	83%	Multi-substance	Participants who received a single, 30-60 minute peer counseling session were more likely to report that they had attended mutual-help group meetings during the first week following detoxification discharge. Trends were also observed: those receiving peer counseling were more likely to remain abstinent from all

		attendance after detoxification discharge.<br>Con: No peer intervention.						substances, and also initiate professional aftercare treatment.
Boisvert et al., 2008	Quasi-experiment	Exp: 'Peer Support Community Program': In a long-term supportive housing community, select individuals are taught to help govern the community and provide ongoing psychosocial support to fellow residents. The Peer Support Community Program aims to help clients maintain abstinence from alcohol and other drugs, and remain in housing, thereby transitioning out of homelessness.<br>Con: A sample of residents living in the same long-term supportive housing community the year prior to instigation of the peer-support program.	Sample: Adults living in permanent supportive housing following inpatient SUD treatment. 100% had a current SUD, 17% had a co-occurring mental illness.<br>Peers: Adults living in permanent supportive housing following inpatient SUD treatment.	*N* = 18<br>(participants' sex not specified)	1, 2, 3, 4, 5, 6, 7, 8, 9, 10, 11, and 12 months	12.5%	Multi-substance	Pre- to post-intervention, participants in the Peer Support Community Program reported more emotional/informational (R^2= 0.39), tangible (R^2= 0.24) and affectionate (R^2= 0.24) support. Participants in the Peer Support Community Program also had lower relapse rates over the study period compared to a sample of residents living in the permanent supportive housing setting the year prior to instigation of the peer-support program.
Smelson et al., 2013	Quasi-experiment	Exp: 'Maintaining Independence and Sobriety through Systems Integration, Outreach, and Networking'	Sample: Military veterans with SUD co-occurring mental disorders who are unemployed and	*N* = 333<br>(F= 4%, M= 96%)	6 and 12 months	70.6%	Multi-substance	In comparison to TAU, those receiving MISSION had greater outpatient session attendance within the 30 days before the 12-

		(MISSION) program, involving a 12-month, intervention developed for military veterans who have experienced homelessness and/or whose ability to return to independent community living is further complicated by co-occurring mental disorders. MISSION includes temporary housing, integrated mental health and SUD treatment delivered via Dual Recovery Therapy (Ziedonis and Stern, 2001), case management, and vocational and peer support.<br><br>Con: Veteran's Administration TAU including temporary housing, medical treatment, consultation with a psychiatrist, group therapy, and vocational training.	have experienced homelessness.<br><br>Peers: Not described.					month follow up (*d*= 1.25), and a greater decline in the number of psychiatric hospitalization nights (*d*= –0.26). Both groups improved on measures of substance use and associated problems at 12 months, with those in MISSION less likely to drink to intoxication (OR= 0.29) and experience serious tension or anxiety (OR= 0.53).
James et al., 2014	Quasi-experiment	Exp: Child welfare substance use treatment program ('Arizona Families FIRST' program), in addition to an enhanced program utilizing trained peer	Sample: Parents or caregivers referred by child protective services to a specialized substance use	*N* = 1,362<br><br>(F= 79%, M= 21%)	36-month consecutive period	32%	Multi-substance	PRSS was associated with faster outreach, and shorter latency to initial clinical assessment (*d*= 0.16), and higher rates of any treatment service initiation compared to

		recovery specialists. Peer recovery coaches provided outreach and engagement to parents recently referred to the program, and helped initiation of SUD treatment. Peer recovery coaches were assigned to a client for approximately 60 days and generally discontinued contact with clients after they had successfully engaged in substance use treatment.<br><br>Con: Child welfare substance use treatment program (‘Arizona Families FIRST’ program) alone.	outpatient treatment program.<br><br>Peers: Parents in recovery from substance use disorder who had achieved reunification and permanent custody of their children following maltreatment allegations.					no peer contact. Those receiving PRSS were less likely to complete treatment, however, among those completing treatment, the average length of treatment was significantly greater for the PRSS + TAU group than controls (*d*= 0.35). Participants receiving PRSS who discontinued treatment remained in treatment longer than controls who discontinued treatment (*d*= 0.36). Groups were not different in terms of total numbers making it to initial assessment appointments, initiating counseling, or discontinuing participation in treatment
Boyd et al., 2005	Single-group retrospective	12 sessions of peer counseling providing psychoeducation about SUD and emotional and informational support to enhance motivation to change substance use behaviors and develop coping strategies for HIV.	Sample: Women with HIV living in rural areas. 100% had substance use problem based on Michigan Alcoholism Screening Test and Drug Abuse Screening Test scores.<br><br>Peers: Not described.	*N* = 13<br><br>(F= 100%)	12 weeks	100%	Multi-substance	No inferential analyses were conducted due to the small sample size. Results however suggest a 12-week peer counseling intervention for substance use may increase participants’ recognition that their alcohol and other drug use is problematic, and increase change behaviors.

Min et al., 2007	Multi-group retrospective	Exp: The ‘Friends Connection Program’: A community-based program in which participants are paired with a peer who has successfully achieved alcohol and other drug abstinence and is successfully coping with their mental health issues. Peer-supports and clients meet approximately once a week for an average of 2 to 5 hours to engage in a variety of community-based activities, including leisure and recreational activities, attend mutual-help groups, and/or spend time talking.<br>Con: A comparable community sample of individuals who did not participate in the ‘Friends Connection Program’.	Sample: Adults identified by the City of Philadelphia that have a history of frequent, long-term, psychiatric hospitalizations. 100% had current alcohol use disorder or other SUD in addition to psychiatric comorbidity.<br>Peers: Individuals with SUD and co-occurring mental disorders who were successfully coping with their mental health issues and had abstained from using alcohol and other drugs for at least three years.	*N* = 484<br>(F= 35%, M= 65%)	N/A	N/A	Multi-substance	Compared to a demographically and diagnostically concordant comparison group, participants in the ‘Friends Connection Program’ had longer periods of living in the community without rehospitalization, and a lower overall number of rehospitalizations over a 3-year period.
Andreas et al., 2010	Single-group retrospective	‘Peers Reach Out Supporting Peers to Embrace Recovery’ (PROSPER): A SUD recovery program based on	Sample: Women and men in SUD recovery who have been incarcerated.<br>Peers: People in SUD recovery who	*N* = 509<br>(F= 32%, M= 68%)	6 and 12 months	Not reported	Multi-substance	From baseline to 12-month assessment, increases in self-efficacy, perceived social support, and quality of life were

		peer-to-peer social support that complements existing services. It includes peer-run groups, coaching, workshop/seminars, social and recreational activities, and community events.	have been incarcerated, plus their families.					observed, as were decreases in perceived stress. Guilt- and shame-based emotions increased over the same period of time.
Armitage et al., 2010	Single-group retrospective	'Recovery Association Project': A community peer recovery service based on leadership training for civic engagement of people in recovery, leading to a range of public and civic involvement among peers.	Sample: Adults in recovery from SUD.<br>Peers: Individuals in recovery from SUD who had completed at least 15 hours each of 'Recovery Association Project' leadership training.	*N* = 152<br>(F= 39%, M= 61%)	6 months	96%	Multi-substance	At 6-month assessment, 86% of clients who had participated in the peer-driven 'Recovery Association Project' indicated no use of alcohol or other drugs in the past 30 days, and another 4% indicated reduced use. 95% reported strong willingness to recommend the program to others, 89% found services helpful, and 92% found materials helpful.
Deering et al., 2011	Single-group prospective	Exp: The 'Mobile Access Project Van': A peer-based mobile service providing a safe place for female sex-workers to rest and eat, and for staff to provide peer-support, condoms and clean syringes, while also acting as a point of contact for referrals to health services.	Sample: Female sex-workers who use alcohol and other drugs.<br>Peers: Not described.	*N* = 242<br>(F= 100%)	N/A	N/A	Multi-substance	Women were more likely to utilize the 'Mobile Access Project Van' if they were at higher risk (i.e., seeing <10 clients per week, and/or working insolated settings; injecting cocaine or injecting/smoking methamphetamine in past 6 months), and were also more likely to access the

		Con: A comparable sample of female sex-workers who did not participate in the 'Friends Connection Program'.						intervention's drop-in center. Past 6-month use of the peer-led outreach program was also associated with a four-fold increase in the likelihood of participants utilizing inpatient SUD treatment including detoxification and residential SUD treatment.
Kelley et al., 2017	Single-group retrospective	'Transitional Recovery and Culture Program': A community-driven, PRSS approach aimed at improving sobriety rates in Native American communities, and increasing community awareness of substance use problems and the need for supporting SUD recovery.	Sample: Adults engaged with tribal chemical dependency programs, tribal health programs, and community social service agencies.<br>Peers: Native Americans recruited from chemical dependency programs.	*N* = 224<br>(F= 51%, M= 49%)	6 months	29%	Multi-substance	At 6-month follow-up, 'Transitional Recovery and Culture Program' participants demonstrated significant reductions from baseline in past 30-day alcohol (d= –0.78), and other drug use (d= –0.64). Participants also endorsed being more concerned about their psychological or emotional problems.
Samuels et al., 2017	Multi-group retrospective	Group 1: 'Lifespan Opioid Overdose Prevention' (LOOP) program: The program provides opioid overdose patients presenting to two hospital emergency departments take-home naloxone, patient education on overdose rescue, and consultation with	Sample: Adults presenting to two hospital emergency departments with opioid overdose.<br>Peers: Recovery coaches in addiction recovery for at least two years who had completed a 36-hour peer recovery coach training program in motivational	*N* = 151<br>(F= 32.5%, M= 67.5%)	12 months	N/A	Opioids	At 12-month follow-up via medical chart review, groups were not significantly different in terms of proportion of participants initiating medication for opioid use disorder, number of times returning to the same emergency department for overdose, number of

		a community-based peer recovery coach for addiction treatment navigation.<br><br>Group 2: Take-home naloxone with print and video patient education materials about naloxone assembly and use, in addition to usual care consisting of medical stabilization and provision of a list of substance use treatment programs in printed discharge instructions.<br><br>Group 3: Usual care only.	interviewing, addiction treatment services, including opioid agonist therapy, and provision of peer-to-peer support.					deaths, and median time to death.
Scott et al., 2018	Single-group retrospective	A combined intervention using peer outreach workers for contacting and identifying out-of-treatment individuals with OUD and a modified version of the 'Recovery Management Checkup' intervention (Scott and Dennis, 2010) that focused only on initial linkage to treatment and engagement.	Sample: Individuals actively using opioids in urban areas identified as high-risk for continued opioid use and overdose.<br><br>Peers: Individuals with a history of opioid use disorder and stable participation in methadone treatment for at least one year.	*N* = 70<br><br>(F= 27%, M= 73%)	30 and 60 days	70%	Opioids	Of participants showing up to the treatment linkage meeting after being approached by peers in natura, 96% were admitted to methadone treatment, with a median time from initial linkage meeting to treatment admission of 2.6 days. 69% were still in treatment 30 days post-intake and 70% at day 60.

Sanders et al., 1998	Cross-sectional	Exp: Peer-led counseling providing comprehensive case management including counseling, support groups, and assistance with housing, transportation, parenting, nutrition and child welfare.<br><br>Con: Counseling from traditionally trained addiction counselors.	Sample: Pregnant and postpartum women in recovery from crack cocaine addiction.<br><br>Peers: Women in recovery from SUD with histories of abusive relationships, homelessness, birth of infants with positive toxicologies, and removal of children by protective services.	*N* = 56<br><br>(F= 100%)	N/A	N/A	Crack cocaine	Clients receiving ongoing counseling from a peer-counselor, compared to clients receiving counseling from traditionally trained addiction counselors were more likely to describe their counselors as empathic, to identify them as the most helpful aspect of the program, to utilize other clinic resources, and to more strongly recommend their program.
Laudet et al., 2016	Cross-sectional	Students residing in college recovery housing at 29 US universities.	Sample: College students in recovery from SUD.<br><br>Peers: Peer-based college recovery support services.	*N* = 486<br><br>(F= 43%, M= 57%)	N/A	N/A	Multi-substance	Abstinent from alcohol and other drugs on average 3 years at the time of the survey, a third of the sample stated they would not be in college were it not for a collegiate recovery program. Top reasons for joining a collegiate recovery program included need for peer recovery support, and wanting to stay abstinent from alcohol and other drugs in the college environment, which is typically not conducive to SUD recovery.

Notes. TAU= treatment as usual; Exp= experimental group, Con= control group; SUD= substance use disorder; AA= Alcoholics Anonymous, NA= Narcotics Anonymous; F= female, M= male; N/A= not applicable
