# Supplementary material for: Lived Experience in New Models of Care for Substance Use Disorder: A Systematic Review of Peer Recovery Support Services and Recovery Coaching
Source: Front Psychol. 2019 Jun 13;10:1052. doi: 10.3389/fpsyg.2019.01052 (PMC6585590; doi:10.3389/fpsyg.2019.01052)
Supplement: Supplementary file 2 [file Data_Sheet_1.pdf]

Appendix A

Systematic Review Search Terms

Pubmed

((“Recovery coaching”[Title/Abstract] OR “Peer recovery support ”[Title/Abstract] OR “Peer-based recovery support services”[Title/Abstract] OR “Individual peer support”[Title/Abstract])) AND ((recovery[Title/Abstract] OR remission[Title/Abstract] OR abstinence[Title/Abstract] OR "harm reduction"[Title/Abstract] OR “substance abuse”[Title/Abstract] OR “substance misuse”[Title/Abstract] OR “substance dependence”[Title/Abstract] OR “drug dependence”[Title/Abstract] OR “substance use disorder”[Title/Abstract] OR “alcohol use disorder”[Title/Abstract] OR “drug use disorder”[Title/Abstract] OR alcohol*[Title/Abstract] OR marijuana[Title/Abstract] OR “THC”[Title/Abstract] OR cannabis[Title/Abstract] OR cocaine[Title/Abstract] OR heroin[Title/Abstract] OR opioid*[Title/Abstract] OR opiate*[Title/Abstract] OR narcotic*[Title/Abstract] OR amphetamine*[Title/Abstract] OR methamphetamine*[Title/Abstract] OR benzodiazepine*[Title/Abstract] OR barbiturate*[Title/Abstract] OR hallucinogen*[Title/Abstract] OR inhalant*[Title/Abstract] OR steroid*[Title/Abstract] OR “club drug*”[Title/Abstract] OR ecstasy[Title/Abstract] OR “MDMA”[Title/Abstract] OR stimulant*[Title/Abstract] OR cost-benefit[Title/Abstract] OR cost-offset[Title/Abstract] OR cost-effectiveness[Title/Abstract]) OR “cost benefit”[Title/Abstract] OR “cost offset”[Title/Abstract] OR “cost effectiveness”[Title/Abstract]))

Embase

(‘Recovery coaching’:ab,ti OR ‘Peer recovery support’:ab,ti OR ‘Peer-based recovery support services’:ab,ti OR ‘Individual peer support’:ab,ti) AND (recovery:ab,ti OR remission:ab,ti OR abstinence:ab,ti OR 'harm reduction':ab,ti OR ‘substance abuse’:ab,ti OR ‘substance misuse’:ab,ti OR ‘substance dependence’:ab,ti OR ‘drug dependence’:ab,ti OR ‘substance use disorder’:ab,ti OR ‘alcohol use disorder’:ab,ti OR ‘drug use disorder’:ab,ti OR alcohol*:ab,ti OR marijuana:ab,ti OR ‘THC’:ab,ti OR cannabis:ab,ti OR cocaine:ab,ti OR heroin:ab,ti OR opioid*:ab,ti OR opiate*:ab,ti OR narcotic*:ab,ti OR amphetamine*:ab,ti OR methamphetamine*:ab,ti OR benzodiazepine*:ab,ti OR barbiturate*:ab,ti OR hallucinogen*:ab,ti OR inhalant*:ab,ti OR steroid*:ab,ti OR ‘club drug*’:ab,ti OR ecstasy:ab,ti OR ‘MDMA’:ab,ti OR stimulant*:ab,ti OR cost-benefit:ab,ti OR cost-offset:ab,ti OR cost-effectiveness:ab,ti OR ‘cost benefit’:ab,ti OR ‘cost offset’:ab,ti OR ‘cost effectiveness’:ab,ti)

CINAHL

AB (“Recovery coaching” OR “Peer recovery support” OR “Peer-based recovery support services” OR “Individual peer support”) AND AB (recovery OR remission OR abstinence OR "harm reduction" OR “substance abuse” OR “substance misuse” OR “substance dependence” OR “drug dependence” OR “substance use disorder” OR “alcohol use disorder” OR “drug use disorder” OR alcohol* OR marijuana OR “THC” OR cannabis OR cocaine OR heroin OR opioid* OR opiate* OR narcotic* OR amphetamine* OR methamphetamine* OR benzodiazepine* OR barbiturate* OR hallucinogen* OR inhalant* OR steroid* OR “club drug*” OR ecstasy OR “MDMA” OR stimulant* OR cost-

benefit OR cost-offset OR cost-effectiveness OR “cost benefit” OR “cost offset” OR “cost effectiveness”)

AB (“Recovery coaching” OR “Peer recovery support” OR “Peer-based recovery support services” OR “Individual peer support”) AND TI (recovery OR remission OR abstinence OR "harm reduction" OR “substance abuse” OR “substance misuse” OR “substance dependence” OR “drug dependence” OR “substance use disorder” OR “alcohol use disorder” OR “drug use disorder” OR alcohol* OR marijuana OR “THC” OR cannabis OR cocaine OR heroin OR opioid* OR opiate* OR narcotic* OR amphetamine* OR methamphetamine* OR benzodiazepine* OR barbiturate* OR hallucinogen* OR inhalant* OR steroid* OR “club drug*” OR ecstasy OR “MDMA” OR stimulant* OR cost-benefit OR cost-offset OR cost-effectiveness OR “cost benefit” OR “cost offset” OR “cost effectiveness”)

TI (“Recovery coaching” OR “Peer recovery support” OR “Peer-based recovery support services” OR “Individual peer support”) AND AB (recovery OR remission OR abstinence OR "harm reduction" OR “substance abuse” OR “substance misuse” OR “substance dependence” OR “drug dependence” OR “substance use disorder” OR “alcohol use disorder” OR “drug use disorder” OR alcohol* OR marijuana OR “THC” OR cannabis OR cocaine OR heroin OR opioid* OR opiate* OR narcotic* OR amphetamine* OR methamphetamine* OR benzodiazepine* OR barbiturate* OR hallucinogen* OR inhalant* OR steroid* OR “club drug*” OR ecstasy OR “MDMA” OR stimulant* OR cost-benefit OR cost-offset OR cost-effectiveness OR “cost benefit” OR “cost offset” OR “cost effectiveness”)

TI (“Recovery coaching” OR “Peer recovery support” OR “Peer-based recovery support services” OR “Individual peer support”) AND TI (recovery OR remission OR abstinence OR "harm reduction" OR “substance abuse” OR “substance misuse” OR “substance dependence” OR “drug dependence” OR “substance use disorder” OR “alcohol use disorder” OR “drug use disorder” OR alcohol* OR marijuana OR “THC” OR cannabis OR cocaine OR heroin OR opioid* OR opiate* OR narcotic* OR amphetamine* OR methamphetamine* OR benzodiazepine* OR barbiturate* OR hallucinogen* OR inhalant* OR steroid* OR “club drug*” OR ecstasy OR “MDMA” OR stimulant* OR cost-benefit OR cost-offset OR cost-effectiveness OR “cost benefit” OR “cost offset” OR “cost effectiveness”)

PsycINFO

Same as for CINAHL
